# Effect of acupoint therapies on chemotherapy-induced nausea and vomiting: A systematic review protocol

**DOI:** 10.1097/MD.0000000000017109

**Published:** 2019-09-13

**Authors:** Jiaqi Hu, Yifeng Shen, Ge Zhang, Jie He, Mingxi Sun, Haishan Zhang, Baojin Hua, Honggang Zheng

**Affiliations:** a Department of Oncology, Guang’anmen Hospital, China Academy of Chinese Medical Sciences; b School of Graduates, Beijing University of Chinese Medicine, Beijing; c Hospital of Chengdu University of Traditional Chinese Medicine, Sichuan, China.

**Keywords:** acupuncture, nausea, neoplasms, systematic review, vomiting

## Abstract

**Background:**

Patients suffering from chemotherapy-induced nausea and vomiting (CINV) might have negative adherence of treatment. Acupoint therapies, including acupuncture, acupressure, acupoints injection, massage, and moxibustion, are safe medical procedures with minimal side effects for CINV, but studies about overall safety and effectiveness of acupoint therapies have not been scientifically and methodically evaluated in recent years. Evaluating the overall safety and effectiveness of acupoint therapies in patients with CINV is the purpose of this review.

**Methods and analysis:**

Relevant randomized controlled trials (RCTSs) are being searched in the following electronic databases: PubMed, Cochrane Library, Web of Science, EMBASE, China National Knowledge Infrastructure (CNKI), Chinese Scientific Journal Database (VIP database), Wanfang Data Knowledge Service Platform, and Chinese Biomedical Literature Database (CBM). We will also attempt to obtain the unpublished academic data by contacting the colleague, professor, or Institute of Traditional Chinese Medicine. The RCTs of the acupoint therapies for CINV patients will be searched in the databases from inception to July 2019. The primary outcomes are defined as severity, duration and frequency of nausea or vomiting, or both. The secondary outcomes are defined as any adverse events and quality of life. Performing the meta-analysis by using RevMan version 5 software. Mean difference (MD) or standardized mean difference (SMD) will express the continuous variables, while relative risk (RR) will express the categorical variables.

**Results:**

The results of this review will provide a high-quality synthesis to evaluate the effectiveness and safety of acupoint therapies for CINV.

**Conclusion:**

This review will provide evidence to estimate whether acupoint therapies are effective interventions for CINV.

**Dissemination:**

Evidence whether acupoint therapies are effective interventions for CINV will be provided by this systematic review. This knowledge will recommend better acupoint therapies and selections of acupoints which might be helpful in treating CINV. The findings of this systematic review will be disseminated via various forms of presentation and publication of the data in a journal or electronic databases.

**PROSPERO registration number:**

CRD42019125538.

## Abstract 2

Supplemental Digital Content is available in the text

**Strengths and limitations**

1. We will objectively evaluate the overall safety and effectiveness of acupoint therapies in CINV management in this systematic review.
2. This systematic review will recommend better acupoint therapies and selections of acupoints in treating CINV.
3. This systematic review will also have some limitations. Different types of acupoint therapies, selections of chemotherapeutics and stage of cancer might run the risk of heterogeneity. Different measurements and tools might lead to inconsistent levels of outcomes. Studies published in English or Chinese was included, which might cause a potential risk of publication bias.

## 1 Introduction

Nausea and vomiting are the most common side effects brought by the chemotherapeutics,^[1]^ which might cause dehydration, electrolyte disorder, malnutrition, and so on, negatively affecting patients’ adherence to treatment.^[2,3]^ Seventy to eighty percent of cancer patients will develop CINV without appropriate antiemetic intervention.^[4]^ Hence, effective management of chemotherapy-induced nausea and vomiting (CINV) is beneficial for patients’ compliance and life quality.

Antiemetics classified into the following categories are recommended for CINV: 5-HT3 serotonin receptor antagonists, tachykinin NK1 receptor antagonist, steroids, olanzapine, dopamine receptor antagonists, and benzodiazepines.^[5]^ However, complete control of CINV remains unsolved.^[6]^ Besides, expensive costs and side effects, including constipation, headaches, hiccups, and so on,^[7]^ of antiemetics also indicate better treatments are still needed to CINV.

Acupoint therapies include acupuncture, acupressure, acupoints injection, massage, and moxibustion, which are safe medical procedures with minimal side effects for CINV. *The National Institutes of Health (NIH)* Consensus Statement has recommended acupoint stimulation as a complementary intervention to prevent CINV.^[8]^ The Oncology Nursing Society also considers acupoint stimulation as a promising intervention for the management of CINV.^[9]^ Previous systematic review has shown that acupuncture-point stimulation would reduce the incidence of acute vomiting.^[10]^ However, we consider it necessary to update it due to its long time of publication.

This systematic review adopts the methods of evidence-based medicine to evaluate the overall safety and effectiveness of acupoint therapies as an additional intervention in CINV management, giving suggestion of acupoint prescription is the further work.

## 2 Methods and analysis

### 2.1 Inclusion criteria for study selection

#### 2.1.1 Types of studies

All the RCTs of acupoint therapies for the management of CINV will be included; the languages of Chinese and English were restricted. Full journal publication with sufficient data for analysis will be required.

#### 2.1.2 Types of patients

Patients suffering from CINV with the following conditions will be included: firstly, definite pathological diagnosis of cancer without restrictions related to type and stage. Secondly, receiving chemotherapy through intravenous injection. And finally, age, gender, race, education status, operation, and types of chemotherapeutics are not restricted. Patients with any of the following conditions will be excluded: firstly, nausea and/or vomiting not caused by chemotherapy, such as metabolic imbalance, mechanical risk factors, intracranial hypertension, and so on. Secondly, serious complications or severe liver/kidney function abnormalities. Thirdly, cognitive communication impairments or mental illness. And finally, pregnant or lactating.

#### 2.1.3 Patient and public involvement

This study is a systematic review, so there are no patients involved.

#### 2.1.4 Types of interventions

##### 2.1.4.1 Experimental interventions

Acupoint therapies, including acupuncture, acupressure, acupoints injection, massage, and moxibustion, will be used with antiemetics in treatment group. Antiemetics applied are the same as the control group. There is no limitation on the materials, types of therapies, selections of acupoints, duration of each treatment, and total courses of treatment. The location of acupoints refers to the WHO standard acupuncture point locations in the Western Pacific Region.^[11]^ All the acupuncturists have obtained acupuncture licenses and receive special training about the trial.

##### 2.1.4.2 Control interventions

Only a standard antiemetic schedule, which is based on the American Society of Clinical Oncology Clinical Practice Guideline,^[12]^ will be received in control group. The types of antiemetics are not restricted.

#### 2.1.5 Outcomes

##### 2.1.5.1 Primary outcomes

The primary outcomes are defined as severity, duration and frequency of nausea or vomiting, or both. Scales or medical or nursing observations will be accepted as the measurement.

##### 2.1.5.2 Secondary outcomes

The secondary outcomes are defined as any adverse events and quality of life, which are measured by validated scales.

### 2.2 Search methods for the identification of studies

RCTSs are being searched in the following electronic databases: PubMed, Cochrane Library, Web of Science, EMBASE, China National Knowledge Infrastructure (CNKI), Chinese Scientific Journal Database (VIP database), Wanfang Data Knowledge Service Platform, and Chinese Biomedical Literature Database (CBM). We will also attempt to obtain the unpublished academic data by contacting the colleague, professor or Institute of Traditional Chinese Medicine. The RCTs of the acupoint therapies for CINV patients will be searched in the databases from inception to July 2019. The strategy will be created according to the Cochrane handbook guidelines. Two reviewers (JQH and YS) will independently search the studies in electronic databases according to the systematic review protocol. The following terms will be used: acupuncture, acupressure, massage, moxibustion, acupoint∗, acupoints injection, acupoint∗ injection, chiropra∗, reflexology, knead, carcinoma, carcinomas, neoplasm, neoplasms, tumor∗, tumour∗, cancer, cancers, vomiting, nausea, nause∗, sickness, vomit∗, emesis, hyperemesis, antiemetic∗, anti-emetic∗. We will use the equivalent search words in Chinese databases. The detailed search strategy for searching the PubMed database is available in Appendix 1.

#### 2.2.1 Searching other resources

We will search for additional trials by examining the reference lists of studies related to acupoint therapies for CINV. We will also search the Clinical Trials.gov and the WHO International Clinical Trials Registry Platform (ICTRP) for new trials relevant to the topic.

### 2.3 Selection of studies

Reviewers will import the literature retrieved to the EndnoteX8 and exclude the duplicate data. Two reviewers (JH and MS) will independently screen titles and abstracts of all included studies referring to the inclusion criteria to exclude irrelevant studies. The remaining studies will be evaluated by reading the full text to confirm the final included studies. Studies with incomplete or questionable information will be decided whether to include or exclude by contacting the corresponding author. Any disagreement arising from this process will be resolved by consensus or a third reviewer (GZ).

### 2.4 Assessment of risk of bias

Two reviewers (GZ and JQH) will evaluate risk of bias in included studies based on the Cochrane collaboration's risk of bias assessment tool. Seven areas will be considered: random sequence generation, allocation concealment, blinding of participants and personnel, blinding of outcome assessment, incomplete outcome data, selective reporting, and other bias. The risk of bias will be categorized as low, unclear and high.^[13]^

### 2.5 Data collection and analysis

#### 2.5.1 Data extraction and management

Two reviewers (YS and HZ) will independently extract the data from each included study using EpiData version 3.1. The information extracted includes: firstly, the basic information of the article (title, author, date of publication, types of study, the number of study groups and center, the sample size, etc). Secondly, methods (randomization, blinding, inclusion and exclusion criteria, study treatment, etc). Thirdly, outcome. Fourthly, methodological quality and hence bias risk. Any disagreement arising from this process will be resolved by consensus or a third reviewer (GZ). We will be in contact with the authors of trials for further information when necessary.

#### 2.5.2 Measurements of treatment effect

Mean difference (MD) or standardized mean difference (SMD) will express the continuous variables, while relative risk (RR) will express the categorical variables. The estimated value and 95% confidence interval (95% CI) of each effect size will be given.

#### 2.5.3 Dealing with missing data

We will attempt to contact the author of the articles to obtain the missing data. We will explain the situation and use the available data to accomplish our analysis if it cannot be obtained.

#### 2.5.4 Assessment of heterogeneity

χ2 test and *I*^2^ statistic test will be used to confirm the heterogeneity of the research results. If the *I*^2^ < 50% or *P* > .10, we will use the fixed-effect model. Otherwise, the random effect model and descriptive analysis will be considered.

#### 2.5.5 Assessment of reporting bias

When more than 5 studies are included, visual dissymmetry on the funnel plot indicates whether there is publication bias. Reporting bias could be also assessed by using Egger's test^[14]^ and Begger's analysis.

#### 2.5.6 Data synthesis

Performing the meta-analysis by using RevMan version 5 software. We will apply the fixed effect model to meta-analysis if there is no obvious heterogeneity among the research results. We will consider the random effect model if there is a level of heterogeneity. Performing the subgroup analysis or meta-regression until the abandonment of the data integration if there is significant heterogeneity.

#### 2.5.7 Subgroup analysis

Subgroup analysis will be necessary if there is significant heterogeneity among the research results. The following items will be considered: firstly, gender, age, and race of patients. Secondly, types and stage of cancer. Thirdly, different chemotherapy drugs. Fourthly, types of acupoint therapies and different selections of acupoints.

#### 2.5.8 Sensitivity analysis

When sufficient trials are available, sensitivity analysis will be performed by sequentially eliding each trial to check the robustness of the final results.

#### 2.5.9 Grading the quality of evidence

We will use the Grading of Recommendations Assessment, Development and Evaluation (GRADE) to evaluate the quality of evidence which is classified into 4 levels: very low, low, moderate, or high.^[15]^

## 3 Discussion

**Figure 1:**
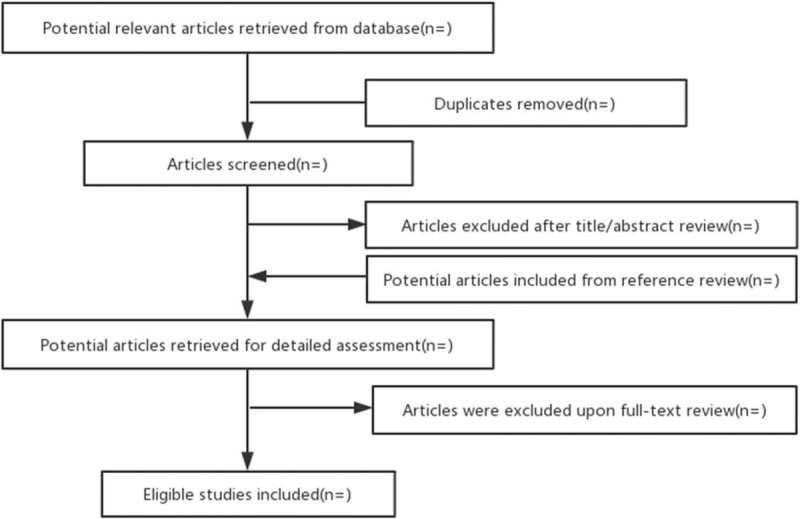
Flow diagram of study selection process.

Acupoint therapies have the characteristics of higher acceptability, less side effect and inexpensiveness. Acupuncture, moxibustion, and auricular acupressure could effectively reduce the symptoms of CINV.^[16–21]^ Taking these studies into consideration, acupoint therapies could be recommended as a nonpharmacological method applied to patients undergoing CINV. But their overall efficacy and safety have not been evaluated scientifically and systematically in recent years. Our study systematically will review currently available researches, with the aim of evaluating the efficacy and safety of the acupoint therapies in patients with CINV. We will also recommend better acupoint therapies and selections of acupoints which might be helpful in treating CINV. The flow chart for this systematic review has been provided in Figure 1.

There are some limitations to this review. Different types of acupoint therapies, selections of chemotherapeutics, and stage of cancer might run the risk of heterogeneity. Different measurements and tools might lead to inconsistent levels of outcomes. In addition, this review will only include studies published in English or Chinese due to the language restriction, which might cause a potential risk of publication bias.

## Author contributions

**Conceptualization:** Jiaqi Hu, Yifeng Shen.

**Data curation:** Ge Zhang, Haishan Zhang.

**Investigation:** Jie He, Mingxi Sun.

**Methodology:** Ge Zhang, Jie He, Mingxi Sun, Haishan Zhang.

**Supervision:** Baojin Hua, Honggang Zheng.

**Writing – original draft:** Jiaqi Hu, Yifeng Shen.

**Writing – review & editing:** Jiaqi Hu.

## Supplementary Material

Supplemental Digital Content
