# Supplementary material for: Effect of acupoint therapies on chemotherapy-induced nausea and vomiting: A systematic review protocol
Source: Medicine (Baltimore). 2019 Sep 13;98(37):e17109. doi: 10.1097/MD.0000000000017109 (PMC6750289; doi:10.1097/MD.0000000000017109)
Supplement: Supplemental Digital Content [file medi-98-e17109-s001.doc]

**Appendix 1.** Search strategy used in PubMed database

| No. | Search items |
| --- | --- |
| 1 | Acupuncture [Mesh Terms] |
| 2 | Acupressure [Mesh Terms] |
| 3 | Massage [Mesh Terms] |
| 4 | Moxibustion [Mesh Terms] |
| 5 | acupuncture [Title/Abstract] |
| 6 | acupressure [Title/Abstract] |
| 7 | acupoint*[Title/Abstract] |
| 8 | acupoints injection [Title/Abstract] |
| 9 | acupoint* injection [Title/Abstract] |
| 10 | massage [Title/Abstract] |
| 11 | moxibustion [Title/Abstract] |
| 12 | chiropra*[Title/Abstract] |
| 13 | reflexology [Title/Abstract] |
| 14 | knead [Title/Abstract] |
| 15 | 1 OR 2 OR 3 OR 4 OR 5 OR 6 OR 7 OR 8 OR 9 OR 10 OR 11 OR 12 OR 13 OR 14 |
| 16 | Carcinoma [Mesh Terms] |
| 17 | [Neoplasms](https://www-ncbi-nlm-nih-gov-cd.vtrus.net/mesh/68009369) [Mesh Terms] |
| 18 | [Neoplasms](https://www-ncbi-nlm-nih-gov-cd.vtrus.net/mesh/68009369)[Title/Abstract] |
| 19 | neoplasm [Title/Abstract] |
| 20 | carcinoma [Title/Abstract] |
| 21 | carcinomas [Title/Abstract] |
| 22 | tumor*[Title/Abstract] |
| 23 | tumour*[Title/Abstract] |
| 24 | cancer [Title/Abstract] |
| 25 | cancers [Title/Abstract] |
| 26 | 16 OR 17 OR 18 OR 19 OR 20 OR 21 OR 22 OR 23 OR 24 OR 25 |
| 27 | vomiting [Mesh Terms] |
| 28 | nausea [Mesh Terms] |
| 29 | nause* [Title/Abstract] |
| 30 | sickness [Title/Abstract] |
| 31 | vomit*[Title/Abstract] |
| 32 | emesis [Title/Abstract] |
| 33 | hyperemisis [Title/Abstract] |
| 34 | antiemetic* [Title/Abstract] |
| 35 | anti‐emetic*[Title/Abstract] |
| 36 | 27 OR 28 OR 29 OR 30 OR 31 OR 32 OR 33 OR 34 OR 35 |
| 37 | 15 AND 26 AND 36 |
| 38 | randomized controlled trial [Publication Type] |
| 39 | controlled clinical trial [Publication Type] |
| 40 | randomized [Title/Abstract] |
| 41 | placebo [Title/Abstract] |
| 42 | drug therapy [Mesh Subheadings] |
| 43 | randomly [Title/Abstract] |
| 44 | trial [Title/Abstract] |
| 45 | groups [Title/Abstract] |
| 46 | 38 OR 39 OR 40 OR 41 OR 42 OR 43 OR 44 OR 45 |
| 47 | animals [Mesh Terms] NOT humans [Mesh Terms] |
| 48 | 46 NOT 47 |
| 49 | 37 AND 48 |
